# In Situ Quantitative Monitoring of Adsorption from Aqueous Phase by UV–vis Spectroscopy: Implication for Understanding of Heterogeneous Processes

**DOI:** 10.1002/advs.202402732

**Published:** 2024-06-23

**Authors:** Xu‐Dan Yang, Bo Gong, Wei Chen, Jie‐Jie Chen, Chen Qian, Rui Lu, Yuan Min, Ting Jiang, Liang Li, Han‐Qing Yu

**Affiliations:** ^1^ CAS Key Laboratory of Urban Pollutant Conversion Department of Environmental Science and Engineering University of Science and Technology of China Hefei 230026 China; ^2^ School of Metallurgy and Environment Central South University Changsha 410083 China; ^3^ School of Environmental and Biological Engineering Nanjing University of Science and Technology Nanjing 210094 China

**Keywords:** adsorption, computational chemistry, heterogeneous reaction, multivariate curve resolution, UV–vis spectroscopy

## Abstract

The development of in situ techniques to quantitatively characterize the heterogeneous reactions is essential for understanding physicochemical processes in aqueous phase. In this work, a new approach coupling in situ UV–vis spectroscopy with a two‐step algorithm strategy is developed to quantitatively monitor heterogeneous reactions in a compact closed‐loop incorporation. The algorithm involves the inverse adding‐doubling method for light scattering correction and the multivariate curve resolution‐alternating least squares (MCR‐ALS) method for spectral deconvolution. Innovatively, theoretical spectral simulations are employed to connect MCR‐ALS solutions with chemical molecular structural evolution without prior information for reference spectra. As a model case study, the aqueous adsorption kinetics of bisphenol A onto polyamide microparticles are successfully quantified in a one‐step UV–vis spectroscopic measurement. The practical applicability of this approach is confirmed by rapidly screening a superior adsorbent from commercial materials for antibiotic wastewater adsorption treatment. The demonstrated capabilities are expected to extend beyond monitoring adsorption systems to other heterogeneous reactions, significantly advancing UV–vis spectroscopic techniques toward practical integration into automated experimental platforms for probing aqueous chemical processes and beyond.

## Graphical abstract

An integrated approach via coupling in situ UV–vis spectroscopy with inverse adding‐doubling method and multivariate curve resolution‐alternating least square is developed to quantify heterogeneous reactions in aqueous solution. It exhibits a great potential toward practical integration into automated experimental platforms as a versatile tool for probing aqueous chemical processes and beyond.

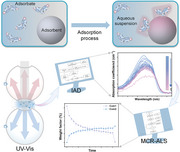

## 1 Introduction

The design of automated in situ platforms capable of precisely revealing heterogeneous processes provides the eye for data‐driven chemical reaction discovery and even brand‐new scientific discipline revelation.^[^ ^1^ ^]^ Adsorption is the fundamental process in various heterogeneous technologies, such as separation, assembly, ion‐exchange, and catalysis.^[^ ^2^ ^]^ Analyzing the adsorption properties of functional materials is critical to clarify their physicochemical science, and ultimately evaluate their performance.^[^ ^3^ ^]^

Research on solute adsorption seems to fall into two distinct directions.^[^ ^4^ ^]^ As the most general ones, solution‐depletion strategies based on batch sampling with offline measurements require neither complex instrumentation nor associated interpretive theory. However, such ex situ methods fail to capture instantaneous information due to lack of temporal resolution.^[^ ^4^, ^5^ ^]^ Moreover, solid‐liquid separation operations such as centrifugation might destroy the weak and loose adsorbed layer, thus propagating a great deal of misinformation in an unpredictable and non‐systematic way. In contrast, some in situ techniques have been developed to track the adsorption process, including ellipsometry, interfacial tensiometry, quartz crystal microbalance, and various types of spectroscopies (such as attenuated total reflectance Fourier transform infrared spectrometer, surface plasmon resonance, surface‐enhanced Raman, and nuclear magnetic resonance (NMR).^[^ ^6^ ^]^ Notably, owing to the ability to probe exchange dynamics over a wide range of timescales with atomic resolution, solution NMR spectroscopy has emerged as a preferred tool to provide structural, thermodynamic, and kinetic information on the sorption equilibria involving multiple adsorbed species and intermediate states.^[^ ^7^ ^]^ Nevertheless, those high‐tech methods usually involve complicated and specific equipment, and require a high‐level of manual operation, making it impractical to be integrated into a closed‐loop autonomous and universal system.^[^ ^5^, ^8^ ^]^ In this regard, it is essential and of great significance to develop efficient and compact in situ techniques with high universality to characterize the aqueous adsorption processes.

UV–vis spectroscopy is the most maturely developed and cost‐effective analytical technique due to its simple equipment, low‐tech operation as well as non‐invasive detection model.^[^ ^9^ ^]^ However, in automated platforms, UV–vis spectroscopy is routinely used to characterize homogeneous systems.^[^ ^10^ ^]^ For heterogeneous systems, the latest autonomous platform integrating UV–vis spectroscopy only achieved qualitative analysis related to spectral peak position changes, band broadening, and intensity variations.^[^ ^11^ ^]^ Recently, UV–vis spectroscopy has been reported to quantify the aqueous adsorption process.^[^ ^12^ ^]^ An universal quantitative method using UV–vis spectroscopy was developed to reveal the protein‐nanoparticle adsorption evolutionary process,^[^ ^12c^ ^]^ while the light scattering interference raised from the nanoparticles to the extinction spectra was ignored.^[^ ^13^ ^]^ We established a method by integrating light scattering correction and nonlinear spectral calibration to quantify the formation of organic pollutants layer onto aqueous suspended microplastics.^[^ ^12d^ ^]^ Nevertheless, due to the demand for prior information on the standard spectra of the adsorbed and aqueous phase molecules, this approach is inefficient and hard to integrate into the autonomous platforms. Hitherto, the potential of the UV–vis spectroscopic module in the automated experimental platforms for quantifying the heterogeneous adsorption process has not been fully utilized.

Multivariate analysis has been widely applied to resolve the chemi‐physical information of pure components in unknown mixtures without needing any prior information.^[^ ^14^ ^]^ Multivariate curve resolution‐alternating least squares (MCR‐ALS) method is among the most widespread ones.^[^ ^15^ ^]^ Spectroscopic datasets can be decomposed through the MCR‐ALS method into a linear combination of a weighted ·set of pure spectral ·profiles, where the weight factor is proportional to their concentrations. MCR‐ALS has been combined with UV–vis spectroscopy in various sceneries,^[^ ^12^, ^16^ ^]^ while there are only a few trials to investigate the heterogeneous processes such as adsorption^[^ ^12^, ^16^ ^]^ and catalysis processes,^[^ ^16e^ ^]^ in which the light scattering interference was ignored or the process was not under suspended conditions. Moreover, the pure spectra of the resolved components identified by MCR‐ALS method should be compared with the reference spectra for chemical evaluation, which are typically recorded from model chemicals in literature or captured via experiments.^[^ ^17^ ^]^ While, it is usually challenging to experimentally capture the reference spectra of the intermediate species of the catalyst and adsorbed species in the heterogeneous processes. Excitingly, benefiting from the development of high‐performance theoretical calculations,^[^ ^18^ ^]^ theoretical molecular calculations might hold a potential to assist the MCR‐ALS method in the spectral signal assignment and chemical structure interpretation,^[^ ^19^ ^]^ especially in high‐throughput autonomous experimental platforms.

Therefore, this work aims to promote the in situ quantitative analysis of the UV–vis spectroscopic technique in the heterogeneous adsorption process in aqueous phase, thereby advancing its practical integration into automated platforms. Through the combination with a one‐step experimental module, the UV–vis spectrophotometer was equipped with an integrating sphere and a two‐step algorithm strategy, namely inverse adding‐doubling (IAD) method for light scattering correction and MCR‐ALS method for spectral deconvolution, to quantify the aqueous adsorption kinetics without prior information of reference spectra. Theoretical calculations were employed to assign the MCR‐ALS solutions to the chemical molecular structural evolution. To the best of our knowledge, it is the first time to combine multivariate analysis with computational spectroscopic simulations under reaction conditions. Thoroughly, the rotational ambiguities of the MCR‐ALS solutions were evaluated through the MCR‐BANDS method (details in Supporting Information). As a case study, bisphenol A (BPA) molecules and polyamide microparticles were respectively selected as the models of adsorbent molecules and adsorbate materials. Finally, the practical applicability of this approach was validated by comparing the adsorption properties of ciprofloxacin hydrochloride (CIP), a typical antibiotic, onto commercial adsorbents such as diatomite and carclazyte.

## 2 Results and Discussion

**Figure 1:**
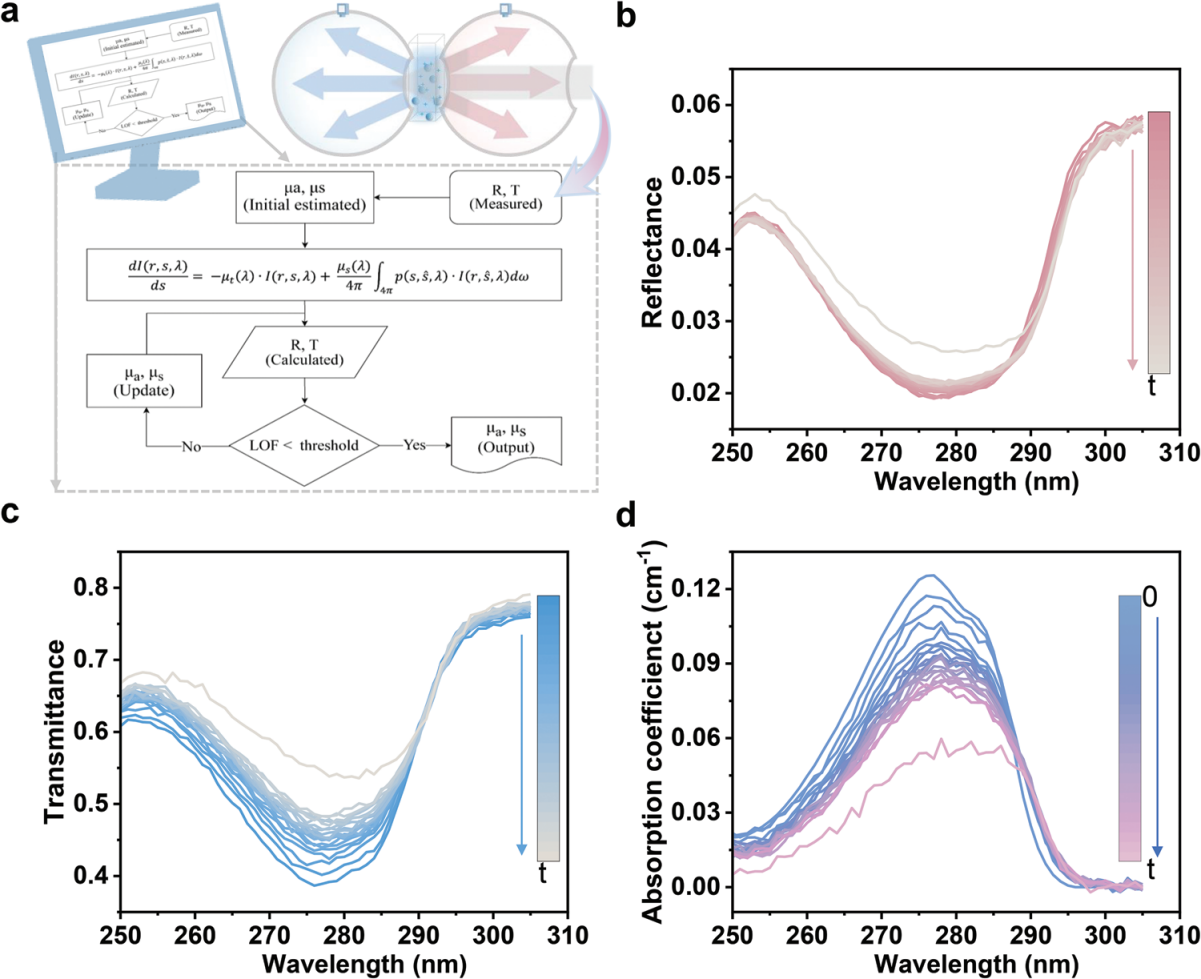
Monitoring of the aqueous adsorption kinetics of BPA (40 mg L^−1^) onto suspended polyamide microparticles (10.45 g L^−1^) in water. a) Schematic of the absorption spectra extraction method. b–d) UV–vis spectral kinetic dataset of b) reflectance, c) transmittance, and d) absorption coefficient monitored 20 times at 1 min time intervals.

The UV–vis absorption spectrum is known as a sensitive indicator for the changes in the surrounding environment of molecules.^[^ ^12^, ^20^ ^]^ Essentially, the aqueous adsorption process is the transfer of molecules from the solution medium to the adsorbent surface. Thus, the adsorption process might be revealed by monitoring the UV–vis absorption spectral kinetics of the suspension. However, the optical properties inherent in a suspension mainly consist of two fractions: the absorption fractions reflecting the electronic energy level structure of the system, and the scattering fractions related to physical collision between photons and particles/molecules depending on the shape and size.^[^ ^21^ ^]^ Notably, adsorbent materials, typically composed of nanometer‐ and micrometer‐sized particles, exhibit strong light scattering effects. Thus, first, the IAD method was adopted to extract the pure optical absorption properties in the aqueous adsorption system, and the scheme is shown in **Figure** **1**a.

The in situ UV–vis total reflectance spectral and total transmittance spectral kinetics of the adsorption of BPA onto suspended polyamide microparticles in water were recorded and calculated (Figure 1b,c). Additionally, the spectroscopic state of the initial BPA solution without adsorbent added and the final adsorption equilibrium state of the suspension were also captured. It is obvious that as the adsorption process proceeded, the intensities of the reflectance and transmittance of the bulk suspension increased. The total transmitted light underwent three types of attenuation: backward light scattering by the polyamide microparticles, light absorption by BPA molecules in the solution, and light absorption by BPA adsorbed on the particles. As the concentration of polyamide microparticles was unchanged, the portion of light scattering remained constant. The adsorption process induced a reduction in the concentration of aqueous BPA molecules, leading to a decrease in light absorption by the solution. Simultaneously, an equal quantity of BPA molecules was transferred to the polyamide microparticle surfaces, resulting in an increase in light absorption. The combined effect led to an increased intensity of total transmittance, and equally, decreased intensity of light absorption, indicating that the aqueous BPA molecule exhibits a higher absorbance compared to the BPA adsorbed on the polyamide microparticles. According to Lambert–Beer's law, these results imply that the molar absorption coefficient of aqueous BPA is larger than that of the adsorbed BPA on the particle. Similarly, the reflected light, generated from the backward scattering of particles, underwent two types of attenuation before reaching the detector: light absorption by BPA molecules in the solution, and light absorption by BPA adsorbed on the particles. The increase in reflectance also demonstrated that the molar absorption coefficient of solution‐phase molecules is larger than that of the aggregated state molecules on the particle, due to the aggregation effect.^[^ ^22^ ^]^

Since the reflectance spectra and total transmittance spectra contain both absorption and scattering signals, they can only be used for the relative quantification of the adsorption process, rather than absolute quantification. After isolating the scattering signals through the IAD method and further processing the “cross‐talk” effect (Figure S1, Supporting Information), the UV–vis absorption coefficient spectra of the suspended adsorption system were extracted (Figure 1d). The spectra exhibited a trend of decreasing intensity (≈60%) along with peak redshift (≈6 nm), indicating an observable spectral change of BPA molecules due to adsorption. Further, the pure UV–vis absorption spectral kinetics reflecting the transfer of the BPA from the aqueous solution into the suspended polyamide microparticles was used for quantification.

**Figure 2:**
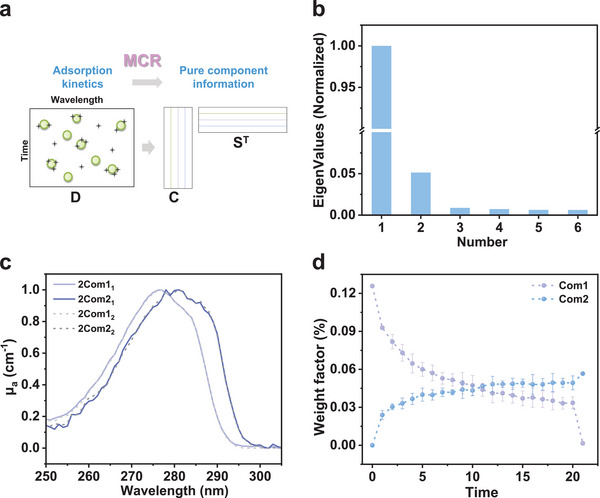
Absorption spectra deconvolution of the kinetic UV–vis absorption spectra. a) Schematic of the MCR‐ALS method. b) Eigenvalues related to the pure species calculated through singular value decomposition. c) Pure spectral profiles obtained by two‐component MCR‐ALS decomposition in two parallel tests. d) Corresponding spectral weight factor obtained by two‐component MCR‐ALS decomposition.

The kinetic optical absorption spectra in a suspension reaction system are the mixture of the evolution signal of their internal chemical species. Therefore, the MCR‐ALS method was applied to identify the spectral profiles of chemical compositions and their corresponding abundance in the time‐evolving UV–vis absorption spectroscopic datasets of the adsorption process. The number of pure components estimated by singular value decomposition, and the initial estimates of the spectra matrix generated by the purest variable detection method, were used as prior information for MCR‐ALS analysis. The eigenvalues related to the chemical components are supposed to be much larger than others like noise. The singular value decomposition analyses indicate that there might be two or three components during the adsorption of BPA onto polyamide microparticles (Scheme S1, Supporting Information; **Figure** **2**b). To determine the pure components during the adsorption process, we performed MCR‐ALS deconvolution in two parallel time‐evolving UV–vis absorption spectroscopic datasets. The pure spectral profiles obtained by two‐component decomposition are consistent in two parallel tests (Figure 2c) while having no parallelism in the three‐component decomposition, especially for the middle component (Figure S2, Supporting Information). Further, in the two‐component case, the convergence was completed within 4 iterations with an *R* ^2^ above 99.95% and a lack of fit below 2.23%. These results indicate that the two‐component MCR‐ALS decomposition fitted well with the experimental data mathematically. Therefore, there were two pure chemical spectral species (Component 2 is on the redshift side of Component 1) in the adsorption system of BPA onto polyamide microparticles, and their corresponding weight factors changing with the adsorption process were also presented (Figure 2d).

MCR methods apply soft constrain for bilinear model solving, making the obtained solution possess more physical meaning and an easier interpretation, at the cost of a certain degree of rotational ambiguities (see details in Supporting Information). This means that an area of feasible solutions can explain the data equally well while fulfilling the same constraints of the system. Due to the inherent defect, the extent of ambiguities should be carefully assessed on the obtained solutions for a critical evaluation of the MCR‐ALS results, like statistical confidence levels evaluation. However, the problem of rotational ambiguity lacks sufficient attention in many studies, thus leading to uncritical results. To understand rotational ambiguity, we conducted MCR‐ALS decomposition on a series of simulated spectral datasets and evaluated the ambiguities of their solutions through the MCR‐BANDS method (see details in Note S1, Supporting Information). It is found that with the increasing overlap in the pure spectra, the rotational ambiguities become heavier (Figure S3, Supporting Information). Thus, in the adsorption process of BPA onto polyamide microparticles, considering the heavier bands overlapping effect in the UV–vis spectroscopy, we evaluated the extent of ambiguities of the MCR‐ALS solutions of the experimental UV–vis absorption spectral dataset through MCR‐BANDS method. The values of the differential signal component contribution function (ΔSCF) were 0.216 and 0.236 for components 1 and 2, respectively, and the areas of feasible solutions for both components were acceptable (Figure S4a, Supporting Information). Notably, if the initial spectrum of the BPA solution and the final spectrum of the suspension in the adsorption equilibrium state were not packed into the kinetic spectral dataset for MCR‐ALS decompositions, the extent of rotational ambiguities as well as the area of feasible solutions of the MCR‐ALS solutions would become much larger (Figure S4b, Supporting Information). The values of ΔSCF increased to 0.3553 for Component 1 and 0.5037 for Component 2. It might be because the spectra of the initial state and equilibrium states of the adsorption suspensions are closer to the spectra of pure components in the adsorption systems. These results indicate that the construction of the spectral dataset would significantly affect the MCR‐ALS solutions.

**Figure 3:**
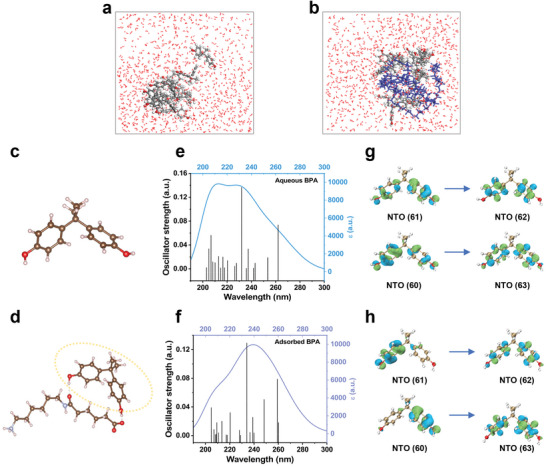
Computational spectroscopic simulation. a,b) Molecular dynamic simulations of 10 BPA molecules in water environments (a) and multiple BPA molecules, polyamide units in water environments (b). c,d) Density functional theory simulation of the free BPA molecules and the optimized BPA‐polyamide structures. e,f) Twenty excitation states and the fitted theoretical UV–vis spectra calculated by time‐dependent density functional theory calculation of aqueous BPA and adsorbed BPA. g,h) Natural transition orbital analysis of aqueous BPA molecules and adsorbed BPA.

Further, theoretical molecular calculations were conducted to assign the spectral signal and interpret the chemical structures of the pure spectral species deconvoluted through the MCR‐ALS method. First, molecular dynamic simulations were performed with the clusters composed of multiple BPA molecules, polyamide units, and water environments to get a macroscopic view of their dynamic interaction during the adsorption process, and the cluster in the absence of polyamide units was set for comparison (see details in Note S2, Supporting Information). The results suggest that BPA significantly interacts with polyamide, likely through van der Waals forces (**Figure** **3**a,b; Figure S5, Supporting Information). Inspired by the molecular dynamic results, the interaction details between BPA and polyamide unit were further investigated through density functional theory simulations (Figure 3c,d). The optimized structures show that after interacting with polyamide, the two molecules bind with each other through hydrogen bonding between the phenolic group in BPA and the carbonyl or carboxyl group in the polyamide unit, as the dashed line shows in Figure 3d. By calculating the atomic Mulliken charge of the BPA‐polyamide complex through density functional theory simulations, it is found that the BPA and polyamide held a charge of +0.068 and −0.068 |e|, respectively, indicating an electron transfer process from BPA to polyamide. Yet further analysis is still required to identify whether the above interaction will lead to a significant change in the UV–vis spectra.

Therefore, twenty excitation states and eventually the theoretical UV–vis spectra of aqueous BPA molecules and adsorbed BPA (the BPA moiety after interacting with polyamide) were calculated by time‐dependent density functional theory calculation (Figure 3e,f). Although the excitation wavelength for the twenty excitation states in adsorbed BPA (Figure 3f) has partial overlapping with those of aqueous BPA (Figure 3e), a significant red‐shift in the theoretical UV–vis spectrum of the adsorbed BPA is demonstrated. Further, the electronic mechanism of the spectral red‐shift effect after adsorption was revealed, including molecular orbital analysis and natural transition orbital (NTO) analysis conducted by the Multiwfn package and Visual Molecular Dynamics software (Figure 3g,h).^[^ ^23^ ^]^ The molecular orbital analysis preliminarily implies a complicated contribution for mixed orbitals caused by the intermolecular interactions (Figure S6, Supporting Information). The NTO contribution results display that NTO 61 to 62 and NTO 60 to 63, which are both attributed to the *π*–π* transition, exhibit >95% of contributions in aqueous BPA and adsorbed BPA (see details in Note S3, Supporting Information). So far, it can be concluded that electrons transfer from the BPA to the polyamide unit, especially for the benzene ring, were responsible for the red‐shift of the UV–vis spectra after BPA adsorbed into polyamide. Up till now, the pure UV–vis absorption spectrum of Component 1 could be assigned to BPA molecules in aqueous solutions and Component 2 to BPA molecules adsorbed in polyamide microparticles.

The efficiency of the proposed method was verified from the perspective of quantification after successfully assigning the two pure spectral profiles to aqueous BPA and adsorbed BPA. The absolute concentration kinetics of aqueous BPA during the adsorption process were calculated from the MCR‐ALS solution of Component 1 based on its molar absorption spectrum (Figure S7, Supporting Information). For reference, the concentrations of the aqueous BPA during the adsorption process were measured through the batch sampling method. With those data in hand, linear regression analysis and Bland‐Altmann plot analysis were conducted to evaluate the quantitative accuracies of the proposed method.

**Figure 4:**
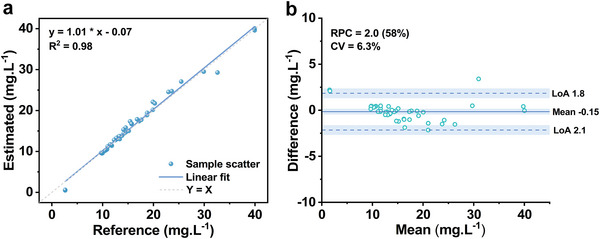
Quantitative performance validation of the aqueous adsorption of BPA onto suspended polyamide microparticles. a) Linear regression analysis comparing the in situ spectral method (estimated) with the ex situ batch sampling method (reference). b) Bland–Altmann plot analysis.

In the linear regression analysis, a significant linear relationship between the reference concentrations and the spectral estimated concentrations is presented (**Figure** **4**a). The root mean square error was 1.0, with a determination coefficient of 0.98 (*P* < 0.001), indicating a high degree of correlation between the proposed method and the traditional isolated‐based method. From the Bland‐Altmann plot, the mean difference was −0.15 mg L^−1^ with a narrow 95% confidence interval, indicating a small difference between the two methods (Figure 4b). Moreover, a normal distribution of the difference was presented as the sample points were uniformly distributed between the limits of agreement (LoA, dash line in Figure 4b, range from −2.1 to 1.8). The estimated repeatability coefficient (RPC) was 2.0 mg L^−1^ with a variation coefficient of 6.3%, suggesting that the absolute difference between any two measurements was estimated to be no >2.0 mg L^−1^ on 95% of occasions. The consistency tests demonstrate that the proposed method exhibited excellent performance in quantifying the heterogeneous adsorption process.

**Figure 5:**
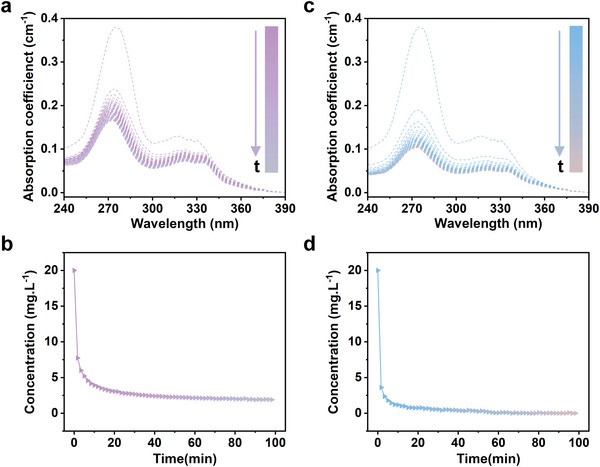
Practical applications of the approach for aqueous adsorption quantification of CIP (20 mg L^−1^) onto commercial adsorbents (200 mg L^−1^). a,c) In situ spectroscopic monitoring of the aqueous adsorption of CIP onto a) diatomite and c) carclazyte. b,d) Adsorption kinetics estimated by the proposed method for the aqueous adsorption of CIP onto b) diatomite and d) carclazyte.

We further verified the practical applicability of our method for screening antibiotic adsorption materials, since the release of excessive antibiotics into the aquatic environment has become a worldwide public health concern due to their potential to accelerate the generation of antibiotic‐resistant bacteria. Thus, it is of great significance to accelerate the discovery of efficient aqueous adsorption materials for antibiotics. The adsorption kinetics of CIP, a typical antibiotic, onto two different kinds of typical commercial adsorbents, i.e., diatomite and carclazyte, were analyzed using the proposed method (**Figure** **5**). The absorption coefficient spectra declined rapidly at the beginning and gradually became stable for both adsorption systems. Moreover, the decrement rate was faster in the diatomite system than that in the carclazyte system. After spectra deconvolution and quantification, the adsorption kinetics show that CIP was adsorbed much quicker onto diatomite than carclazyte. The pseudo‐second‐order model was found to explain the adsorption kinetics effectively. For diatomite, the determined pseudo‐second‐order rate constant was 0.019 min g mg^−1^, and the equilibrium adsorption capacity was 99 mg g^−1^.^[^ ^24^ ^]^ For carclazyte, the determined pseudo‐second‐order rate constant was 0.009 min g mg^−1^, and the equilibrium adsorption capacity was 90.9 mg g^−1^. The great fitness for the pseudo‐second‐order model indicates that the rate‐limited step in the uptakes of CIP was the chemisorption process. It demonstrates that the adsorption rates depended on the adsorption capacity of the adsorbents, rather than the concentration of the CIP. Therefore, diatomite was a superior adsorbent for CIP in aquatic environments.

It should be noted that the applicability of this approach still has some constraints. The adsorbate molecules should have characteristic UV–vis spectral signals, such as conjugated molecules or transition metal complexes with *π*→π*, *n*→π*, or *d*→*d* transition structures.^[^ ^19^, ^25^ ^]^ The basis of our method to resolve the spectra of adsorbed molecules from those of free‐state molecules is the shifts in spectral peaks and/or changes in intensity resulting from the interaction between molecules and particles after adsorption. If the adsorbed molecules themselves have characteristic sharp peaks, the spectral differences between the adsorbed and free‐state molecules will be more significant. Consequently, our method can better resolve them and quantitatively determine their concentrations at a certain adsorption time. However, if the spectral features of the adsorbed molecules are broad bands, it poses a greater challenge for spectral decomposition compared to characteristic sharp bands, because the broad bands in the UV–vis spectrum may lead to ambiguity in chemical structural identification. Despite these limitations, this efficient and concise approach can significantly improve the integration and versatility of UV–vis spectroscopic technology into closed‐loop autonomous experimental platforms, and advance its capability for the elucidation of complex heterogeneous processes in the aqueous phase in a time‐resolved manner after incorporation with fiber optics‐equipped spectrometers.

## 3 Conclusion

In summary, we have successfully advanced the UV–vis spectroscopic technique to quantify the heterogeneous adsorption kinetics in aqueous phase in situ. By combining with the IAD method for light scattering correction, the MCR‐ALS method for spectral deconvolution, and the theoretical spectroscopic calculations for pure spectral assignment, the proposed method is able to quantify the aqueous adsorption kinetics successfully without prior information for reference spectra. Inspiringly, the molecular theoretical simulations of dynamic property and electronic structure excellently connect MCR‐ALS solutions with chemical molecular structural evolution. Notably, the construction of the spectral dataset would significantly influence the extent of rotational ambiguities of the MCR‐ALS solutions. Such an efficient and concision approach is expected to be applicable in other heterogeneous reaction systems and presents a promising advance in transforming UV–Vis spectroscopic techniques into automated experimental platforms for elucidating complex aqueous chemical processes. The authors have cited additional references within the Supporting Information.

## Conflict of Interest

The authors declare no conflict of interest.

## Supporting information

Supporting Information. Additional references were cited in Supporting Information.[26]

## Data Availability

The data that support the findings of this study are available from the corresponding author upon reasonable request.

## References

[1] a) Q. Zhu , F. Zhang , Y. Huang , H. Xiao , L. Zhao , X. Zhang , T. Song , X. Tang , X. Li , G. He , B. Chong , J. Zhou , Y. Zhang , B. Zhang , J. Cao , M. Luo , S. Wang , G. Ye , W. Zhang , X. Chen , S. Cong , D. Zhou , H. Li , J. Li , G. Zou , W. Shang , J. Jiang , Y. Luo , Natl. Sci. Rev. 2022, 9, nwac190; 36415316 10.1093/nsr/nwac190 PMC9674120

[2] a) D. M. Ruthven , Principles of adsorption and adsorption processes, John Wiley & Sons, New York 1984;

[3] a) E. Perez‐Botella , S. Valencia , F. Rey , Chem. Rev. 2022, 122, 17647; 36260918 10.1021/acs.chemrev.2c00140 PMC9801387

[4] a) E. A. Vogler , Biomaterials 2012, 33, 1201; 22088888 10.1016/j.biomaterials.2011.10.059 PMC3278642

[5] I. Holland , J. A. Davies , Front. Bioeng. Biotechnol. 2020, 8, 571777. 33282848 10.3389/fbioe.2020.571777 PMC7691657

[6] a) B. L. Mojet , S. D. Ebbesen , L. Lefferts , Chem. Soc. Rev. 2010, 39, 4643; 20949193 10.1039/c0cs00014k

[7] a) K. Tedsree , C. W. Chan , S. Jones , Q. Cuan , W. K. Li , X. Q. Gong , S. C. Tsang , Science 2011, 332, 224; 21474757 10.1126/science.1202364

[8] a) M. Seifrid , R. Pollice , A. Aguilar‐Granda , Z. Morgan Chan , K. Hotta , C. T. Ser , J. Vestfrid , T. C. Wu , A. Aspuru‐Guzik , Acc. Chem. Res. 2022, 55, 2454; 35948428 10.1021/acs.accounts.2c00220 PMC9454899

[9] a) H.‐H. Perkampus , UV‐VIS Spectroscopy and its applications, Springer Science & Business Media, Berlin, Germany 2013;

[10] G. Wang , B. Xin , G. Wang , Y. Zhang , Y. Lu , L. Guo , S. Li , C. Chen , X. Cheng , J. Ma , ChemRxiv 2021.

[11] Y. Jiang , D. Salley , A. Sharma , G. Keenan , M. Mullin , L. Cronin , Sci. Adv. 2022, 8, eabo2626. 36206340 10.1126/sciadv.abo2626 PMC9544322

[12] a) B. L. Darby , B. Auguié , M. Meyer , A. E. Pantoja , E. C. Le Ru , Nat. Photonics 2015, 10, 40;

[13] S. Nawalage , P. Wathudura , A. Wang , M. Wamsley , S. Zou , D. Zhang , Anal. Chem. 2023, 95, 1899. 10.1021/acs.analchem.3c00823 PMC12823024 37382879

[14] A. de Juan , R. Tauler , Anal. Chim. Acta 2021, 1145, 59. 33453882 10.1016/j.aca.2020.10.051

[15] a) J. Jaumot , A. de Juan , R. Tauler , Chemom. Intell. Lab. Syst. 2015, 140, 1;

[16] a) F. Sales , M. Callao , F. Rius , Chemometr. Intell. Laboratory Syst. 1997, 38, 63;

[17] J. Felten , H. Hall , J. Jaumot , R. Tauler , A. de Juan , A. Gorzsas , Nat. Protoc. 2015, 10, 217. 25569330 10.1038/nprot.2015.008

[18] a) S. Ye , G. Zhang , J. Jiang , Proc. Natl. Acad. Sci. USA 2021, 118, 2025879118; 10.1073/pnas.2025879118 PMC8256048 34185681

[19] a) K. Hemelsoet , Q. Qian , T. De Meyer , K. De Wispelaere , B. De Sterck , B. M. Weckhuysen , M. Waroquier , V. Van Speybroeck , Chemistry 2013, 19, 16595; 24281808 10.1002/chem.201301965

[20] K. Kemnitz , N. Tamai , I. Yamazaki , N. Nakashima , K. Yoshihara , J. Phys. Chem. 1986, 90, 5094.

[21] S. A. Prahl , M. J. van Gemert , A. J. Welch , Appl. Opt. 1993, 32, 559. 20802725 10.1364/AO.32.000559

[22] a) G. V. Barnett , W. Qi , S. Amin , E. N. Lewis , V. I. Razinkov , B. A. Kerwin , Y. Liu , C. J. Roberts , J. Phys. Chem. B 2015, 119, 15150; 26563591 10.1021/acs.jpcb.5b08748

[23] a) T. Lu , F. Chen , J. Comput. Chem. 2012, 33, 580; 22162017 10.1002/jcc.22885

[24] S. Peng , Y. Wei , Y. Huang , L. Wei , P. Chen , Environ. Sci. Pollution Res. 2023, 30, 98490. 10.1007/s11356-023-29217-x 37608178

[25] R. A. Schoonheydt , Chem. Soc. Rev. 2010, 39, 5051. 21038052 10.1039/c0cs00080a

