# Supplementary material for: In Situ Quantitative Monitoring of Adsorption from Aqueous Phase by UV–vis Spectroscopy: Implication for Understanding of Heterogeneous Processes
Source: Adv Sci (Weinh). 2024 Jun 23;11(32):2402732. doi: 10.1002/advs.202402732 (PMC11348127; doi:10.1002/advs.202402732)
Supplement: Supplementary file 1 — Supporting Information. Additional references were cited in Supporting Information.[26] [file ADVS-11-2402732-s001.docx]

Supplementary Information for

**In-Situ Quantitative Monitoring of Adsorption from Aqueous Phase by UV-Vis Spectroscopy: Implication for Understanding of Heterogeneous Processes**

Xu-Dan Yang^[a]^, Bo Gong^[a]^, Wei Chen*^[b]^, Jie-Jie Chen^[a]^, Chen Qian^[a]^, Rui Lu^[c]^, Yuan Min^[a]^, Ting Jiang^[a]^, Liang Li^[a]^, Han-Qing Yu*^[a]^

^a^CAS Key Laboratory of Urban Pollutant Conversion, Department of Environmental Science and Engineering, University of Science and Technology of China, Hefei, Anhui 230026, China

^b^School of Metallurgy and Environment, Central South University, Changsha, Hunan 410083, China

^c^School of Environmental and Biological Engineering, Nanjing University of Science and Technology, Nanjing, 210094, China

**Contents**

Supplementary Methods

Supplementary Notes 1 to 3

Supplementary Figures 1 to 7

Supplementary Tables 1 and 2

Supplementary References

1. **Supplementary Methods**

**Measurement setups**

UV-Vis spectrophotometer (Lambda 650s, PerkinElmer Inc., USA) equipped with a 60-mm integrating sphere was used to collect the total transmittance and total reflectance spectra. The suspensions were placed in a quartz glass cuvette, customized to be 5 mm (light path length) × 40 mm (width) × 50 mm (height), for spectra measurements. A homemade stirring device was used to mix the suspension in the cuvette. The adsorption processes were also quantified by solution-depletion methods, centrifugation isolation followed with an ultra-performance liquid chromatography test (UPLC, LC-16, Shimadzu Co., Japan), for reference.

**Experimental design**

The adsorption process of BPA onto polyamide microparticles (70±30 μm in diameter, purchased from Sinopec Maoming Petrochemical Co. China) in water was used as a case study to verify the accuracy of the proposed method. The reflectance and transmittance spectral kinetics of the adsorption process was monitored 20 times at 1 min time intervals. Each spectrum was collected from 250 nm to 325 nm at 1 nm wavelength with an integration time of 0.2 s. In addition, the spectroscopic state of the initial BPA solution without adsorbent added and the final adsorption equilibrium state of the suspension were also recorded. For comparison, the concentrations of the aqueous BPA in the supernatant during the adsorption process were measured by batch sampling method (isolated via centrifugal at 10000 g at the same time interval with spectra measurements) and then analyzed using the UPLC test. Each experiment was performed in ultra-pure water and was conducted twice. For practical applications, the adsorption properties of CIP onto diatomite and carclazyte were investigated using the proposed method. The spectral kinetics of the adsorption processes were monitored at time intervals of 100 s for 60 times.

**Inverse adding-doubling (IAD) method for absorption spectra extraction**

Radiative transfer theory describes light propagation through a complex medium and taking place in the form of absorption and scattering, which are characterized by absorption coefficient (μ_a_) and scattering coefficient (μ_s_)^[1]^. The integrating spheres measurement coupled with the IAD method is recognized as the “gold standard” to separate the μ_a_ and μ_s_ in different complex heterogenous systems with great variance in particle sizes and shapes^[2]^. The bulk optical properties, absorption coefficient and scattering coefficient, were estimated through iterative inversion of the radiative transfer equations (equation (1) and (2)) and were resolved by the IAD method.

$$\begin{aligned} \frac{dI\left( \boldsymbol{r,s},\lambda\right)}{d\boldsymbol{s}}= -\mu_{t}\left( \lambda\right)\cdot I\left( \boldsymbol{r,s},\lambda\right)+\frac{\mu_{s}\left( \lambda\right)}{4\pi}\int_{0}^{4\pi} \boldsymbol{p}\left( \boldsymbol{s,}\hat{\boldsymbol{s}},\lambda\right)\cdot\boldsymbol{I}\left( \boldsymbol{r},\hat{\boldsymbol{s}},\lambda\right)d\omega\#\left( 1 \right) \end{aligned}$$

$$\begin{aligned} \mu_{t}\left( \lambda\right)=\mu_{a}\left( \lambda\right)+\mu_{s}\left( \lambda\right) \#\left( 2 \right) \end{aligned}$$

where $\boldsymbol{p}\left( \boldsymbol{s,}\hat{\boldsymbol{s}},\lambda\right)$ is the phase function, $\omega$ is the solid angle, $I(r,\boldsymbol{s},\lambda)$ is the light intensity at a distance ***r*** from the light source along with the directional vector **s**, $\mu_{t}\left( \lambda\right)$, $\mu_{a}\left( \lambda\right)$, $\mu_{s}\left( \lambda\right)$ are the total extinction coefficient, absorption coefficient, and scattering coefficient (cm^-1^).

Briefly, the radiative transfer equation was first used to calculate the reflectance (R) and transmittance (T) for a single “infinitesimally” thin sample layer. Then, this layer was “doubled” to calculate the R and T of the doubled layer. This process of doubling was repeated until the desired thickness of the homogeneous sample is reached. Finally, the IAD algorithm estimated the *μ*_a_ and *μ*_s_ by iteratively changing their values in the AD simulations. The optimization was stopped when the relative difference in the standard deviations of the residuals between the experimental R/T and the reproduced data through the adding-doubling method in two consecutive iterations is below a threshold value. In the IAD program, the refractive index of the sample was set to equal to the refractive index of water, 1.33, and the value of the anisotropy factor, *g*, was fixed to 0.9. Other parameters of the IAD procedures are given in Table S1.

If the sample characterizes strong light absorption, the absorption and scattering coefficient would be poorly separated, resulting in a so-called “cross-talk” effect^[3]^. Herein, a new strategy was proposed to eliminate the “cross-talk” effect in equation (3). As the concentrations of particles kept the same during the adsorption process, the light path length as well as the scattered light were considered kept consistent. The absorption interference of the adsorbents was also removed simultaneously. Finally, the obtained pure absorption coefficient spectra were processed by baseline correction.

$$\begin{aligned} \mu_{a_{molecule}}=\mu_{t_{suspension}}-\mu_{t_{adsorbent}} \#\left( 3 \right) \end{aligned}$$

where $\mu_{a_{molecule}}$is the absorption coefficient of molecules in the suspension, $\mu_{t_{adsorbent}}$ is the extinction coefficient of pure adsorbent, and $\mu_{t\_suspension}$ is the extinction coefficient of the suspension.

**Multivariate curve resolution-alternating least squares (MCR-ALS) method analysis for absorption spectra deconvolution**

The time-evolving or spatially distributed experimental spectroscopic datasets could be decomposed by the MCR-ALS method into a weighted set of pure spectral profiles^[4]^, where the weight factor is proportional to their concentrations, under the condition of a bilinear model written as:

$$\begin{aligned} D=C*S^{T}+E=\sum_{n =1}^{N} c_{n}s_{n}^{T}+E=D^{*}+E\#\left( 4 \right) \end{aligned}$$

where D (ns*nl dimension, ns spectra measured at nl wavelength) represents the time-dependent spectra dataset, the column profiles of matrix C and the row profiles of S^T^ are the concentrations and pure spectral profiles respectively of the resolved components. E is the residual matrix not explained by the numerical model and should be close to the experimental error ideally.

The kinetic UV-Vis absorption spectroscopic datasets of the adsorption process were deconvoluted to differentiate the spectra and concentrations profiles of pure chemical species by MCR-ALS GUI 2.0 toolbox, a GUI updated version for the MCR-ALS algorithm under MATLAB. Each resolved pure contribution was thus represented by a pure spectral signature (a row in S^T^) and a related abundance (a column in C). The related tutorials and code are available on the MCR-ALS webpage (www.mcrals.info). The non-negativity of the concentration and pure spectra were set as the constraints. The threshold value was set as 0.1. The pure spectra were normalized by spectra of equal height. The Flow chart of the MCR-ALS method is as follows:


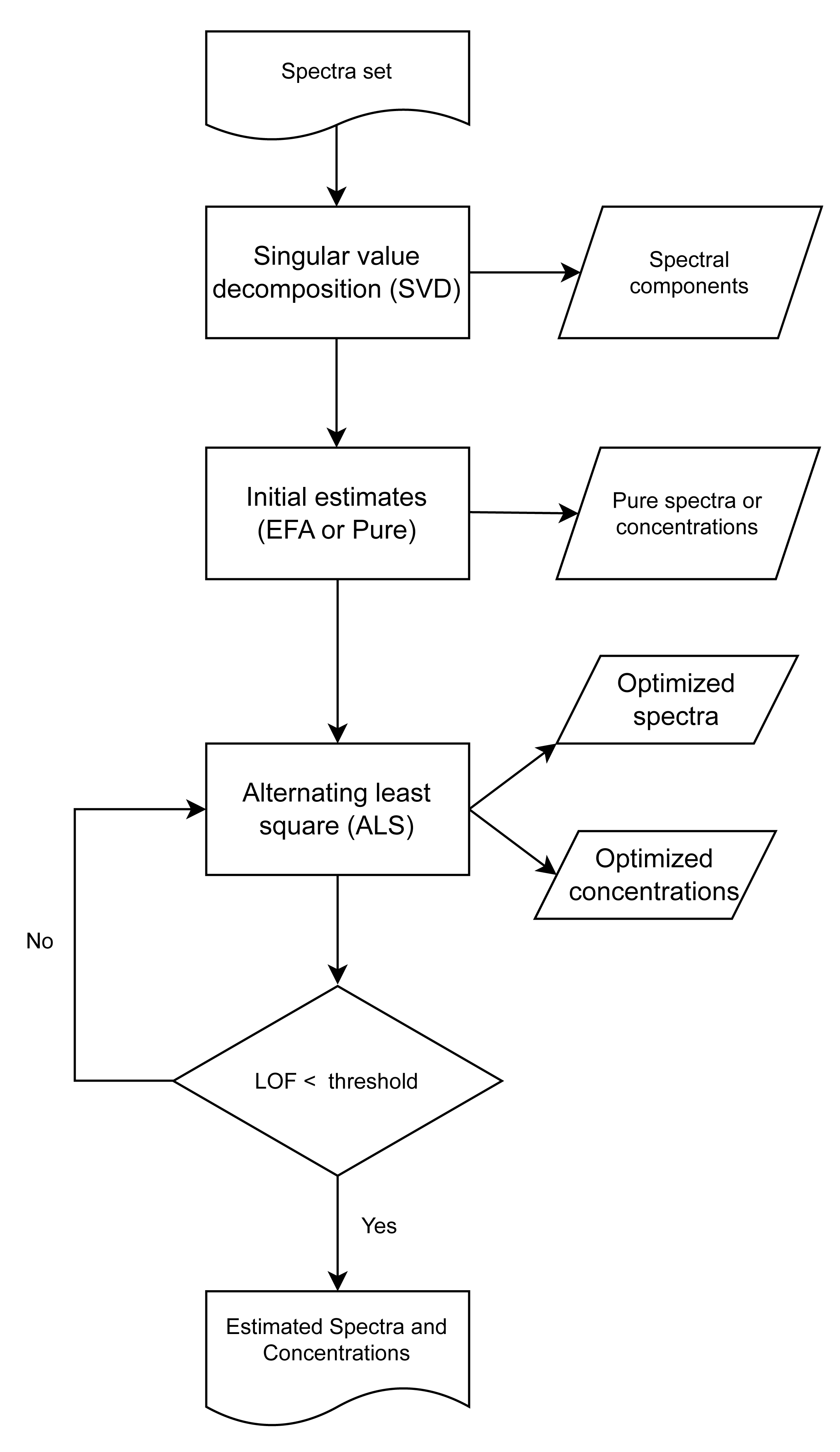


**Rotational ambiguity evaluation**

Mathematically, for any non-singular matrix T, the identity matrix $I=TT^{-1}$ can be inserted in the MCR-ALS calculations as equation (5), thus resulting in a certain degree of rotational ambiguities^[5]^. MCR-BANDS method evaluates the extension of rotational ambiguity for each resolved component by calculating the minimization ($\mathrm{SCF}^{\min}$) and maximization ($\mathrm{SCF}^{\max}$) signal component contribution function (SCF) values, defined as equation (6)^[6]^. The value of $\mathrm{SCF}_{n}$ ranges from 0 to 1. $\mathrm{SCF}^{\max}$ and $\mathrm{SCF}^{\min}$illustrate the boundary of the feasible solutions. When the difference ($\Delta SCF$) between $\mathrm{SCF}_{n}^{min}$ and $\mathrm{SCF}_{n}^{max}$ is close to 0, it means less ambiguity for component n. A graphical user interface program with MATLAB, MCR-BANDS GUI, can be downloaded on the MCR-ALS webpage (www.mcrals.info).

$$\begin{aligned} D=CS^{T}+E=CTT^{-1}S^{T}+E=C_{new}S_{new}^{T}+E \#\left( 5 \right) \end{aligned}$$

$$\begin{aligned} {SCF}_{n}=\frac{\left\| c_{n}s_{n}^{T} \right\|}{\left\| CS^{T} \right\|} \#\left( 6 \right) \end{aligned}$$

where $c_{n}$ and $s_{n}$ are the *n*^th^ column and row of concentration profile $C$ and pure spectra profile $S^{T}$ matrices, ${SCF}_{n}$is the relative signal contribution of a particular component *n* to the whole signal for the mixture of N components (*n* = 1,…, N).

The absolute concentrations of the pure components were calculated from the MCR-ALS solution according to the following equation defined as:

$$\begin{aligned} c_{n}=\frac{\omega_{n}s_{n}^{T}}{\varepsilon_{n}^{T}} \#\left( 7 \right) \end{aligned}$$

where for the n^th^ component, $c_{n}$ is the absolute concentration, $s_{n}^{T}$ and $\omega_{n}$are the normalized pure spectrum and the corresponding weight factor deconvoluted from the MCR-ALS method, $\varepsilon_{n}^{T}$ is the molar absorption spectrum.

**Spectroscopic simulation for pure spectra assignment**

The molecular dynamic (MD) simulations for the cluster composed of ten BPA molecules and polyamide with ten units were performed using the Forcite module in the Materials Studio package. The other case with only ten BPA molecules was set for comparison. The residue volume besides the BPA or polyamide molecules was filled with water molecules to simulate the solution environment. For MD simulations, the Universal forcefield was applied^[7]^. The Ewald and Atom-based methods were selected to describe the electrostatic and van der Waals interactions^[8]^. The cluster was geometrically optimized with a Smart algorithm, followed by the ensemble NVT and NVE for 2 ns each^[9]^. The temperature was kept at 298.15 K with the Nosé method^[9a]^. The structures after the NVE ensemble were obtained, and the corresponding trajectory files were further analyzed including the mean square displacement (MSD) and radial distribution function (RDF).

All the density functional theory (DFT) calculations were performed using Gaussian 16 software^[10]^. The free BPA and BPA- polyamide complex were constructed and optimized with B3LYP/6-31++G(d,p) level^[11]^. The BPA moiety after interacting with PA, regarded as adsorbed BPA, was fixed followed by the single point energy calculation. The DFT-D3 method and Polarizable Continuum Model (PCM) were applied for the correction of dispersion forces and solvation effects, respectively^[12]^. The frequencies were also calculated together with the optimization to ensure there are no imagery values.

As for the free BPA and adsorbed BPA, the excitation properties (twenty excitation states) and theoretical UV-Vis spectra were also obtained through time-dependent DFT (TD-DFT) calculation in Gaussian 16. The molecular orbital (MO) and natural transition orbital (NTO) analysis were conducted with Multiwfn package and Visual Molecular Dynamics (VMD) software^[13]^.

**Adsorption kinetic model**

The pseudo-second-order kinetic equation is expressed as follows:

$$\begin{aligned} \frac{t}{q_{t}}=\frac{1}{k_{2}q_{e}^{2}}+\frac{t}{q_{e}} \#\left( 8 \right) \end{aligned}$$

where $t$ is the reaction time (min), $q_{t}$ (mg$\cdot$g^-1^) is the amount of BPA or p-Np adsorbed at nylon 66 microplastics at time t, $q_{e}$ is the equilibrium adsorption capacity (mg$\cdot$g^-1^), and $k_{2}$ is the pseudo-second-order rate constant (mg$\cdot$g^-1^$\cdot$min^-1^).

Using the equation above, ${t/q}_{t}$ as a function of *t* was plotted and linearly fitted. The second-order rate constant $k_{2}$ and equilibrium adsorption capacity $q_{e}$ could be calculated with the intercept $1/k_{2}q_{e}^{2}$ and the slope ${1/q}_{e}$.

**Statistical analysis**

The lack of fit (LOF) and the explained data variance (R^2^) are defined as below:

$$\begin{aligned} LOF=\sqrt{\sum_{i,j} \frac{e_{i,j}^{2}}{\sum_{i,j} d_{i,j}^{2}}} \#\left( 9 \right) \end{aligned}$$

$$\begin{aligned} R^{2}=\left( 1-\sqrt{1-LOF} \right) \#\left( 10 \right) \end{aligned}$$

where *d* and *e* refer to an element of the experimental data matrix and the associated residual value respectively.

The coefficient of determination (R-squared) and root mean square error (RMSE) are defined as:

$$\begin{aligned} R\text{-}suqared=1-\frac{RSS}{TSS} \#\left( 11 \right) \end{aligned}$$

$$\begin{aligned} RMSE=\frac{\sqrt{\sum_{i=1}^{n} {(Estimated}_{i}-{{Reference}_{i})}^{2}}}{n} \#\left( 12 \right) \end{aligned}$$

where RSS is the residual sum of squares and TSS is the total sum of squares, n is the number of samples, estimated and reference are the calculated concentration in the proposed method and measured concentration through the traditional isolated method, respectively.

In the Bland-Altmann (B-A) plot, the limits of agreement (LoA), coefficient of variation (CV) and reproducibility coefficient (RPC) are defined as:

$$\begin{aligned} LoA=\mu\pm1.96*\frac{\delta}{\sqrt{n}} \#\left( 13 \right) \end{aligned}$$

$$\begin{aligned} CV=\frac{\delta}{\mu}*100\% \#\left( 14 \right) \end{aligned}$$

$$\begin{aligned} RPC=1.96*\sqrt{2}*\frac{\delta}{\sqrt{n}} \#\left( 15 \right) \end{aligned}$$

where $\mu$ is the mean value, $\text{δ}$ is the standard deviation, and n is the number of samples.

The performance of the MCR-ALS solution was assessed by two parameters: LOF and R^2^. The accuracy of quantification was evaluated through linear regression analysis and B-A plot analysis. In the linear regression analysis, the R-squared is equal to the square of the sample correlation coefficient, reflecting the regression efficiency of the method. RMSE is the standard deviation of the errors, reflecting the quantitative accuracy of the method. B-A plot showed the difference against the mean between the two methods and was used to assess the consistency between two quantitative measurements^[14]^. LOA assessed the level of agreement between the two measurement methods. CV and RPC reflected the dispersion degree of two groups of data. MATLAB software was employed for statistical analyses.

1. **Supplementary Notes**

**Supplementary Note 1**

To obtain an understanding of rotational ambiguity, we conducted MCR-ALS decomposition on a series of simulated spectral datasets of two-component systems and evaluated the ambiguities of their solutions through the MCR-BANDS method (Fig. S3). It is found that as the overlap of the two pure spectra increases, the differential signal contribution function ($\Delta SCF$) value changes from near 0 to above 0.6 (Fig. S3c), indicating heavier rotational ambiguities. Taking three datasets D_1_, D_2,_ and D_3_ as examples (shown in Fig. S3e), as the pure components spectra of the two components get closer (from S_1_^T^ to S_3_^T^), the signal feasible solution range of the concentration profile (blue lines in Fig. S3g) become much larger for both components, while the MCR-ALS solution (red lines) is proved to be only one of the possible solutions.

**Supplementary Note 2**

Firstly, MD simulations were performed with the clusters composed of multiple BPA molecules, polyamide units, and water environments to get a macroscopic view of their dynamic interaction during the adsorption process, and the cluster in the absence of polyamide units was set for comparison. In the optimized structures of BPA and BPA-polyamide clusters, both 10 free BPA molecules and the complex clusters show a relative gathering instead of uniformed disperse into the whole box systems (Figs. 3a and 3b). Further, the MSD analysis for aqueous BPA and BPA-polyamide with the same time scale was performed (Fig. S5a). On the one hand, the higher MSD values indicate the more rapid diffusion rate of aqueous BPA. On the other hand, the similar changing tendency of BPA and polyamide moiety in the BPA-polyamide structure suggests that these two sections moved synchronously. In addition, the RDF shows that the distance between BPA and polyamide parts is mainly located at around 5 angstroms (Fig. S5b), which is likely to form van der Waals interaction. Briefly, those results reveal that the BPA interacts significantly with polyamide.

**Supplementary Note 3**

Among all the excitation states, S7 (the seventh excitation state counted from the one with the lowest energy) in both cases showed the strongest signals and thus was recorded as a representative. The MO contribution for this state S0 to S7 in aqueous and adsorbed BPA was collected (Table S2 and Fig. S7). Both cases displayed a complicated contribution resulting from multiple molecular orbitals, containing the highest occupied molecular orbitals (HOMO), lowest unoccupied molecular orbitals (LUMO), and the adjacent orbitals. Compared with the aqueous BPA, the transition of the adsorbed BPA involved a more complex molecular orbital contribution, implying the influence caused by the BPA-PA interactions. In addition, among those MO transition contribution values, the highest contribution was only 46.47% and 23.67%, some orbitals even lead to negative effects. No group holds a dominant contribution (at least 80%). Therefore, another orbital analysis is necessary to clarify the transition mechanisms. Further, the NTO contribution results display that the couple of NTO 61 to 62 and NTO 60 to 63 has a contribution of 49.90% and 53.12% in aqueous BPA and adsorbed BPA, respectively. The latter couple has a contribution of 46.54% and 42.30% in aqueous BPA and adsorbed BPA, respectively. Summing these two couples, more than 95% of contributions are exhibited.

1. **Supplementary Figures**

**
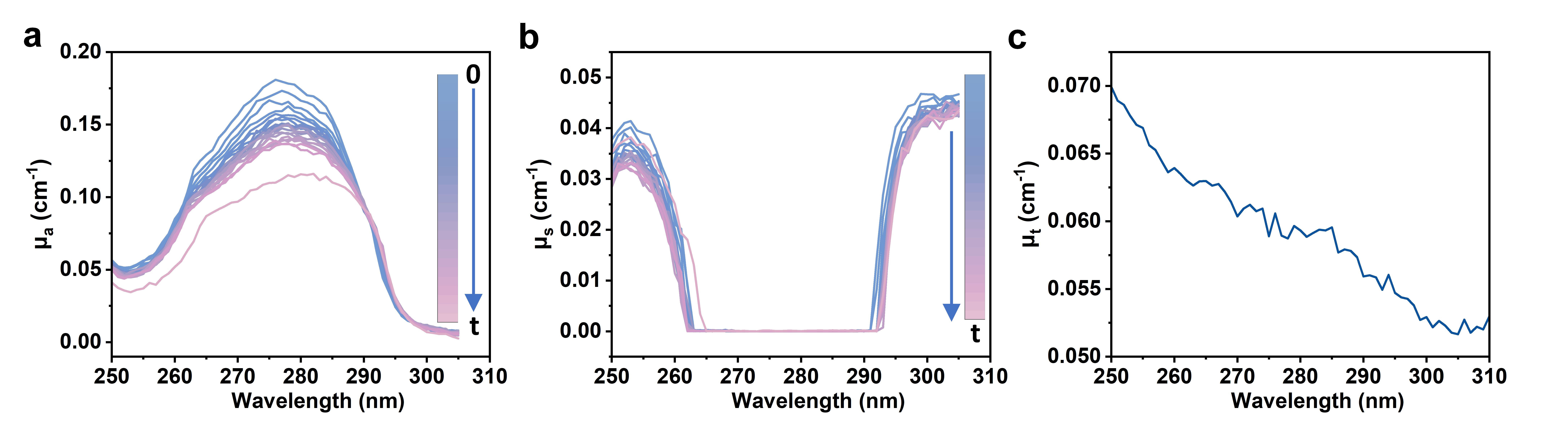
**

**Fig. S1 |** Monitoring of the adsorption kinetics of Bisphenol A (40 mg.L^-1^) onto suspended polyamide microparticles (10.45 g.L^-1^) in water: (a) estimated absorption coefficient spectra and (b) estimated scattering coefficient spectra; (c) extinction coefficient of suspended polyamide microparticles (10.45 g.L^-1^) in water.


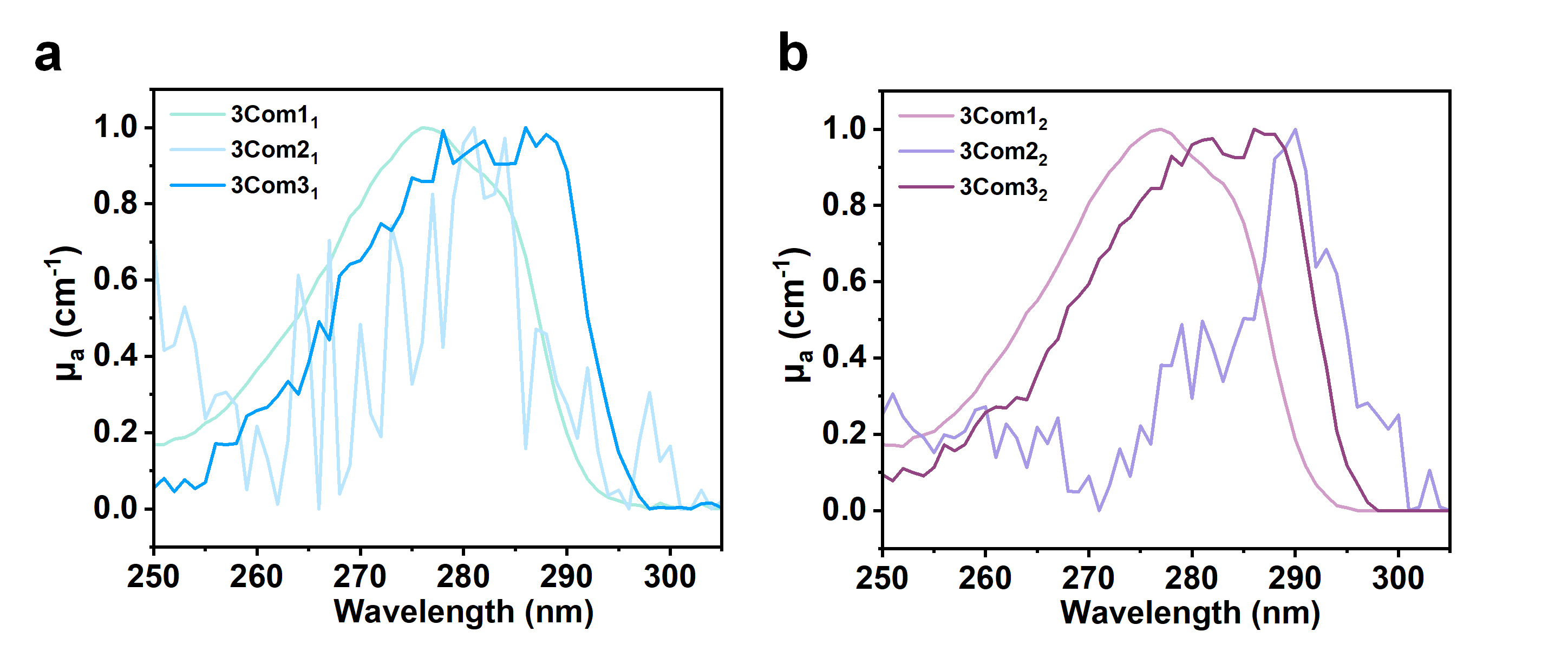


**Fig. S2 |** Normalized pure spectral profiles obtained by three-component MCR-ALS decomposition in two parallel tests.

**

**

**Fig. S3 |** MCR-ALS decomposition on simulated kinetic spectral datasets of the two-component system: (a) whole spectral profiles (the standard deviation of the Gaussian curve is 15, the mean value is 0 for component 1, and the mean is selected sequentially between -50 and -1 for component 2); (b) concentration profiles; (c) differential signal contribution function ($\Delta SCF$) values. Three representative datasets with different degrees of spectral overlap and their corresponding signal feasible solution ranges: large degrees of peak overlap D_1_ (d_1_, e_1_, f_1_, g_1_); middle degrees of peak overlap D_2_ (d_2_, e_2_, f_2_, g_2_); and small degrees of peak overlap D_3_ (d_3_, e_3_, f_3_, g_3_).

**
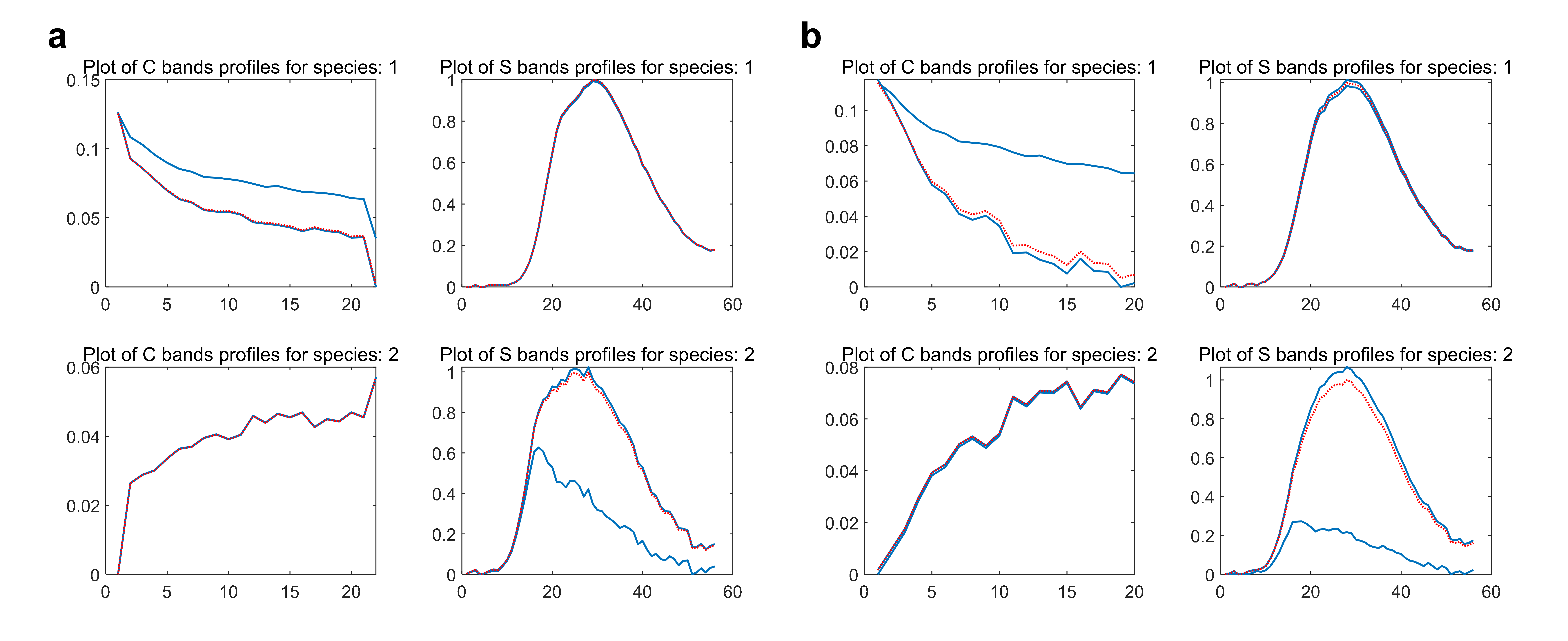
**

**Fig. S4 |** The feasible solution ranges of MCR-ALS concentration and spectra solutions of the experimental UV-Vis absorption spectral dataset of the adsorption process (a) with and (b) without the initial spectrum of the BPA solution and the final spectrum of the suspension in the adsorption equilibrium state packed into the kinetic spectral dataset for MCR-ALS decomposition.

**
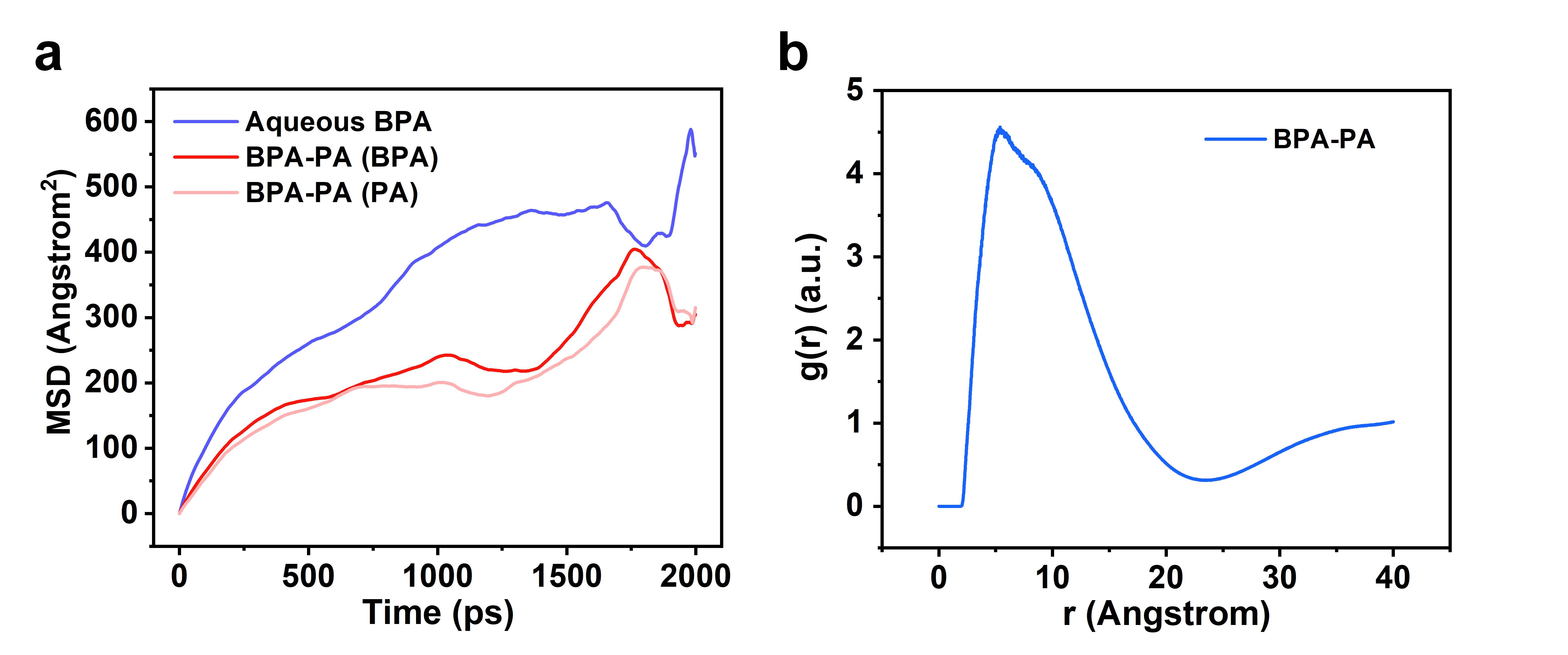
**

**Fig. S5 |** Molecular dynamic (MD) simulations of the BPA and polyamide system: (a) optimized structures of 10 BPA molecules and water environments; (b) optimized structures of multiple BPA molecules, polyamide units, and water environments; (c) mean square displacement (MSD) analysis with the same time scale; (d) radial distribution function (RDF).


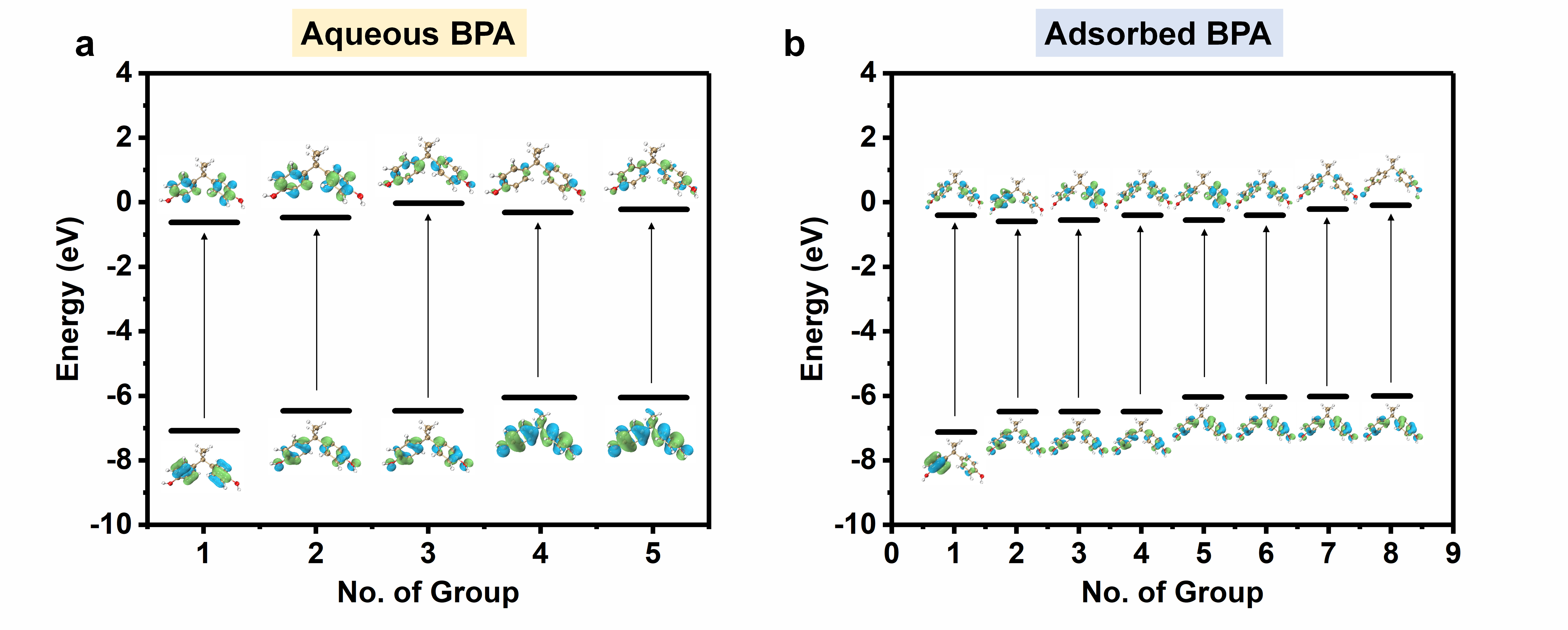


**Fig. S6 |** The schematic of MO transition involved in S0 to S7: (a) aqueous BPA and (b) adsorbed BPA. S0 and S7 represent the ground state and the seventh excitation state.


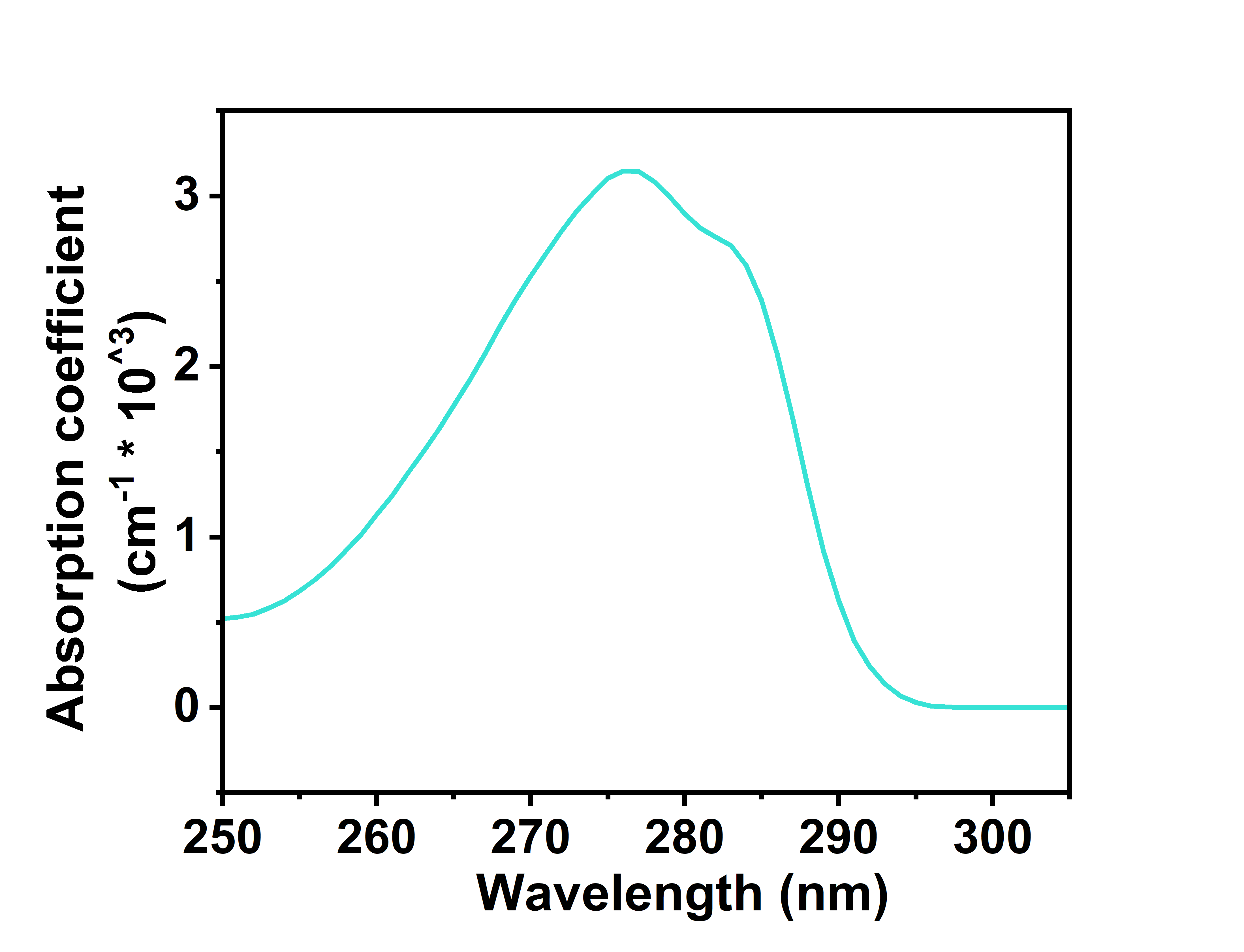


**Fig. S7 |** The molar UV-Vis absorption spectrum of aqueous BPA molecules.

1. **Supplementary Tables**

**Table S1 |** Parameters for the IAD Programs.

| Properties of the sample and measurements setup |  |
| --- | --- |
| Index of refraction of the sample | 1.33 |
| Thickness of sample (mm) | 5 |
| Thickness of slides (mm) | 1.25 |
| Diameter of illumination beam (mm) | 3.24 |
| Index of refraction of the top and bottom slides | 1.45 |
| Reflectivity of the reflectance calibration standard | 0.97 |
| Properties of the sphere used for reflection measurements |  |
| Sphere Diameter (mm) | 60 |
| Sample Port Diameter (mm) | 22.5 |
| Entrance Port Diameter (mm) | 22.5 |
| Detector Port Diameter (mm) | 7 |
| Reflectivity of the sphere wall (mm) | 0.97 |
| Properties of the sphere used for transmission measurements |  |
| Sphere Diameter (mm) | 60 |
| Sample Port Diameter (mm) | 22.5 |
| Entrance Port Diameter (mm) | 22.5 |
| Detector Port Diameter (mm) | 7 |
| Reflectivity of the sphere wall | 0.97 |
| Number of measurements (R,T) | 2 |

**Table S2 |** Contributions of different molecular orbitals for the S7 state.

| Structure | MO transition | Contribution (%) |
| --- | --- | --- |
| Aqueous BPA | HOMO-2 → LUMO | 2.19 |
|  | HOMO-1 → LUMO+1 | 46.47 |
|  | HOMO-1 → LUMO+5 | 2.48 |
|  | HOMO → LUMO+2 | -10.38 |
|  | HOMO → LUMO+3 | 29.54 |
| Adsorbed BPA | HOMO-2 → LUMO+2 | -2.04 |
|  | HOMO-1 → LUMO | 23.67 |
|  | HOMO-1 → LUMO+1 | -7.38 |
|  | HOMO-1 → LUMO+2 | 11.07 |
|  | HOMO → LUMO+1 | 3.52 |
|  | HOMO → LUMO+2 | 6.82 |
|  | HOMO → LUMO+3 | 20.03 |
|  | HOMO → LUMO+4 | -14.23 |
|  | HOMO-2 → LUMO+2 | -2.04 |
|  | HOMO-1 → LUMO | 23.67 |

**Supplementary References**

[1] C. F. Bohren, D. R. Huffman, *Absorption and scattering of light by small particles*, John Wiley and Sons, New York, **1983**.

[2] a) S. A. Prahl, *Oregon Medical Laser Center, St. Vincent Hospital* **2011**, *1*, 1-74; b) J. W. Pickering, S. A. Prahl, N. Vanwieringen, J. F. Beek, H. J. C. M. Sterenborg, M. J. C. Vangemert, *Appl. Opt.* **1993**, *32*, 399-410.

[3] a) E. Zamora-Rojas, B. Aernouts, A. Garrido-Varo, D. Pérez-Marín, J. E. Guerrero-Ginel, W. Saeys, *Innovative Food Sci. Emerging Technol.* **2013**, *19*, 218-226; b) X. He, X. Fu, X. Rao, Z. Fang, *Postharvest Biol. Technol.* **2016**, *121*, 62-70.

[4] a) J. Felten, H. Hall, J. Jaumot, R. Tauler, A. de Juan, A. Gorzsas, *Nat. Protoc.* **2015**, *10*, 217-240; b) S. Li, Y. Hu, A. Li, J. Lin, K. Hsieh, Z. Schneiderman, P. Zhang, Y. Zhu, C. Qiu, E. Kokkoli, T. H. Wang, H. Q. Mao, *Nat. Commun.* **2022**, *13*, 5561; c) A. Martini, E. Borfecchia, *Crystals* **2020**, *10*.

[5] A. Golshan, H. Abdollahi, S. Beyramysoltan, M. Maeder, K. Neymeyr, R. Rajko, M. Sawall, R. Tauler, *Anal. Chim. Acta* **2016**, *911*, 1-13.

[6] a) J. Jaumot, R. Tauler, *Chemom. Intell. Lab. Syst.* **2010**, *103*, 96-107; b) J. Jaumot, A. de Juan, R. Tauler, *Chemom. Intell. Lab. Syst.* **2015**, *140*, 1-12; c) X. Zhang, Z. Zhang, R. Tauler, *Talanta* **2019**, *202*, 554-564.

[7] A. K. Rappé, C. J. Casewit, K. Colwell, W. A. Goddard III, W. M. Skiff, *J. Am. Chem. Soc.* **1992**, *114*, 10024-10035.

[8] P. P. Ewald, *Ann. Phys* **1921**, *369*, 1-2.2.

[9] a) S. Nosé, *MolPh* **1984**, *52*, 255-268; b) J. R. Ray, *CoPhR* **1988**, *8*, 109-151.

[10] M. e. Frisch, G. Trucks, H. Schlegel, G. Scuseria, M. Robb, J. Cheeseman, G. Scalmani, V. Barone, G. Petersson, H. Nakatsuji, Gaussian, Inc., Wallingford CT, **2016**.

[11] a) K. L. Schuchardt, B. T. Didier, T. Elsethagen, L. Sun, V. Gurumoorthi, J. Chase, J. Li, T. L. Windus, *Journal of chemical information and modeling* **2007**, *47*, 1045-1052; b) A. D. Becke, *The Journal of Chemical Physics* **1993**, *98*, 5648-5652.

[12] a) S. Grimme, J. Antony, S. Ehrlich, H. Krieg, *J. Chem. Phys.* **2010**, *132*; b) S. Miertuš, E. Scrocco, J. Tomasi, *ChPh* **1981**, *55*, 117-129.

[13] a) T. Lu, F. Chen, *J. Comput. Chem.* **2012**, *33*, 580-592; b) W. Humphrey, A. Dalke, K. Schulten, *J. Mol. Graphics* **1996**, *14*, 33-38.

[14] a) J. M. Bland, D. G. Altman, *Int. J. Nurs. Stud.* **2010**, *47*, 931-936; b) D. Giavarina, *Biochem. Med.* **2015**, *25*, 141-151.
